# Optical Imaging of Magnetic Particle Cluster Oscillation and Rotation in Glycerol

**DOI:** 10.3390/jimaging7050082

**Published:** 2021-04-29

**Authors:** River Gassen, Dennis Thompkins, Austin Routt, Philippe Jones, Meghan Smith, William Thompson, Paul Couture, Dmytro A. Bozhko, Zbigniew Celinski, Robert E. Camley, Guy M. Hagen, Kathrin Spendier

**Affiliations:** 1 BioFrontiers Center, University of Colorado Colorado Springs, Colorado Springs, CO 80918, USA; egassen@uccs.edu (R.G.); dthompk2@uccs.edu (D.T.); aroutt@uccs.edu (A.R.); pjones5@uccs.edu (P.J.); msmith29@uccs.edu (M.S.); wthompso@uccs.edu (W.T.); zcelinsk@uccs.edu (Z.C.); rcamley@uccs.edu (R.E.C.); ghagen@uccs.edu (G.M.H.); 2 Department of Physics and Energy Science, University of Colorado Colorado Springs, Colorado Springs, CO 80918, USA; pcouture@uccs.edu (P.C.); dbozhko@uccs.edu (D.A.B.)

**Keywords:** barium hexaferrite, iron oxide, viscosity, magnetic particle clusters, drug delivery

## Abstract

Magnetic particles have been evaluated for their biomedical applications as a drug delivery system to treat asthma and other lung diseases. In this study, ferromagnetic barium hexaferrite (BaFe${}_{12}$O${}_{19}$) and iron oxide (Fe${}_{3}$O${}_{4}$) particles were suspended in water or glycerol, as glycerol can be 1000 times more viscous than water. The particle concentration was 2.50 mg/mL for BaFe${}_{12}$O${}_{19}$ particle clusters and 1.00 mg/mL for Fe${}_{3}$O${}_{4}$ particle clusters. The magnetic particle cluster cross-sectional area ranged from 15 to 1000 μm${}^{2}$, and the particle cluster diameter ranged from 5 to 45 μm. The magnetic particle clusters were exposed to oscillating or rotating magnetic fields and imaged with an optical microscope. The oscillation frequency of the applied magnetic fields, which was created by homemade wire spools inserted into an optical microscope, ranged from 10 to 180 Hz. The magnetic field magnitudes varied from 0.25 to 9 mT. The minimum magnetic field required for particle cluster rotation or oscillation in glycerol was experimentally measured at different frequencies. The results are in qualitative agreement with a simplified model for single-domain magnetic particles, with an average deviation from the model of 1.7 ± 1.3. The observed difference may be accounted for by the fact that our simplified model does not include effects on particle cluster motion caused by randomly oriented domains in multi-domain magnetic particle clusters, irregular particle cluster size, or magnetic anisotropy, among other effects.

## 1. Introduction

Excessive mucus production is a significant cause of airway obstruction and mortality in over 24 million US asthma patients [1]. Asthma and related illnesses represent approximately tens of billions in healthcare costs annually. Although there are existing drugs that limit excessive mucus production, there is no cure for excessive mucus production in asthma and other chronic lung diseases. This is likely due to the complex mucus structure that forms a physical barrier, preventing drugs from reaching the diseased airway mucosa (epithelium) [2,3,4]. Nanoparticle systems have been previously investigated for their potential in treating asthma and other lung diseases [4,5]. For instance, to overcome mucus clearance and guide the therapeutics through the mucus barrier, magnetic iron oxide (Fe${}_{3}$O${}_{4}$) nonaparticles [4] and barium hexaferrite (BaFe${}_{12}O_{19}$) nanoparticles [6] have been used previously. For example, barium hexaferrite nanoparticles coated with an anti-inflammatory drug were pulled by external magnetic forces through the mucosal barrier [6]. These experiments showed that barium hexaferrite nanoparticles can indeed penetrate a ∼100 μm thick mucus layer in a static magnetic field gradient of 360 T/m. However, the penetration time of barium hexaferrite nanoparticles for a 100 μm thick mucus layer was up to 30 min, which is too long for this technology to be applied to drug delivery through mucus barriers. Therefore, it has been proposed that magnetic particles with the ability to physically oscillate and rotate may decrease the mucus barrier penetration time by effectively cutting through the mucus mesh [6]. The work presented here investigates the magnetic field requirements for particle rotation at given frequencies and fluid viscosities.

Magnetic particles have been investigated for their magnetic properties and the ability to control their oscillation and rotation through a magnetic field [7,8]. For this reason, they have been studied for various biomedical applications, including drug delivery systems, by using time-varying magnetic fields [9,10,11,12,13,14,15,16]. Magnetic barium hexaferrite (BaFe${}_{12}O_{19}$) nanoparticles have been investigated in high-frequency applications due to their high magnetic anisotropy, among other reasons [17]. In other works, magnetic particles have been studied for targeting and destroying cancer cells [18,19,20,21]. Most in vitro studies were performed in low-viscosity fluids, which ranged from $10^{-4}$ to $10^{-3}$ Pa·s, such as water, model bodily fluids, cell culture fluids, or alcohol [6,7,9,11,12,17,18,22,23,24]. In this paper, magnetic particle oscillation and rotation was investigated in glycerol with a significantly higher viscosity, i.e., ∼1 Pa·s. Glycerol’s viscosity is within the viscosity range of human mucus. Human mucus viscosity values can be as high as $10^{4}$–$10^{6}$ Pa·s and as low as $10^{-2}$ Pa·s [25].

In the experiments presented here, multi-domain magnetic particle clusters were subjected to low driving frequencies that ranged from 10 to 180 Hz. Rotating and oscillating fields were produced by wire spools inserted into a light microscope used to visualize particle cluster motion. The applied magnetic field magnitude was as high as 9 mT. The collected images were then processed in ImageJ and MATLAB. to extract the observed particle cluster oscillation or rotation frequencies. The measured frequency of the particle cluster rotation or oscillation was then compared to a simplified model that estimated the required magnetic field to rotate a single-domain magnetic particle in fluids of different viscosities. The minimum magnetic field strength predicted by the simplified model was in a qualitative agreement with the experiments and within an average deviation of 1.7 ± 1.3. Water was used as a test fluid to verify the experimental conditions. In this experiment, we did not investigate properties such as the phase lag of particle magnetization compared to the applied time-varying field. A discussion of this topic can be found in Reference [24].

## 2. Materials and Methods

### 2.1. Magnetic Particle Solution

For this project, we used commercially available magnetic particles. Magnetic particle samples in 100% glycerol or water were prepared by mixing barium hexaferrite powder containing particles that were less than 500 nm in size (polyhedral-shaped from Nanostructured and Amorphous Materials, Inc., Katy, TX, USA), at a concentration of 2.5 mg/mL or 50–100 nm iron oxide (Fe${}_{3}$O${}_{4}$) nanopowder (Sigma-Aldrich, MilliporeSigma, St. Louis, MO, USA, product number 637106) at a concentration of 1.0 mg/mL in a 1.5 mL micro-centrifuge tube. To reduce the size of the barium hexaferrite particles, the raw powder was hand-ground with a mortar and pestle for two hours. The FeNP concentration in glycerol and water samples was lowered compared to 2.5 mg/mL for BaPs due to FeNPs’ higher tendency to cluster or clump together, making it more difficult to isolate single FeNPs and measure their rotation and oscillation frequencies. The samples were placed in a bath sonicator for ten minutes to break apart very large agglomerates or clusters. Before creating each sample from the BaP or FeNP mixtures, the solution, which was stored in a centrifuge tube, was mixed with a vortexer. Sample slides for optical imaging were created by stacking four SecureSeal™ slides (Sigma-Aldrich, MilliporeSigma, St. Louis, MO, USA, product number GBL654008) on a piece of cover glass. The SecureSeal™ slide imaging well had a diameter of 9 mm and a thickness of 0.12 mm. Cover glass slips (Fisher Scientific, Atlanta, GA, USA, product number 50-311-65) were 18 × 18 mm${}^{2}$ squares with a thickness from 0.13 to 0.17 mm. The BaP mixture or the FeNP mixture was pipetted into a 9 mm well, and the second piece of cover glass was placed over top to seal the sample chamber.

### 2.2. Magnetic Particle Characterization

The crystallographic structure of the magnetic particles was evaluated using an X-ray diffraction (XRD) system (Rigaku SmartLab 5, Rigaku Innovative Technologies, Inc., Auburn Hills, MI, USA). The magnetic properties were studied using a superconducting quantum interference device (SQUID) (SQUID Magnetometer, Quantum Design, San Diego, CA, USA). The mass magnetization was measured in the temperature range of 298 to 340 K in an applied field of 1 T. The hysteresis loops were measured at room temperature, 298 K, up to 5 T.

A scanning electron microscope (SEM) (Tuscan Vega3, Tescan Analytics, Fuveau, France) was used to obtain detailed images of the particle clusters. First, a carbon conductive tape (PELCO Tabs, 16084-1, Ted Pella, Inc., Redding, CA, USA) was attached to a removable SEM imaging stage. Then, approximately 10 μL of BaPs in water at a concentration of 2.5 mg/mL or FeNPs in water at a concentration of 1.0 mg/mL were pipetted onto the carbon tape. Next, the sample was allowed to air dry for ten minutes before being sputter-coated with gold (Denton Vacuum Desk V, Denton Vacuum, Inc., Moorestown, NJ, USA) and imaged with the SEM. The sputtered gold layer was approximately 5 nm.

### 2.3. Wire Coil Microscope Insert

**Figure 1:**
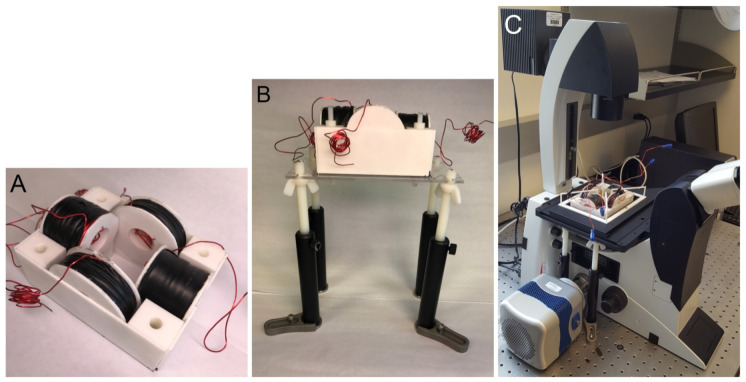
(**A**) Two pairs of wire spools in a perpendicular arrangement housed in a casing. The assembly is 13 cm long and 9.5 cm wide. (**B**) The platform that holds the wire coil assembly. (**C**) Entire coil insert with a sample holder inserted into a Leica DMI3000 microscope.

Two sets of wire spools in a quadrupole configuration can be used to control magnetic particle cluster oscillation and rotation [26]. To image the particle cluster motion with an optical microscope, a microscope insert was engineered to fit within the stage of Lecia’s DMI6000 and DMI3000 inverted microscopes (Leica Microsystems Inc., Buffalo Grove, IL, USA). The insert was designed based on the following strict and adjustable parameters. The strict parameters included the insert dimensions, opening for samples to be imaged, and required magnetic field magnitude. The insert must allow imaging with 10×, 20×, or 40× objectives. Specifically, the coil insert must fit within a 6.2 inch × 4.0 inch opening and allow for movement of the sample, which is placed in the center of the spools in the horizontal plane (x-y plane) of the stage. A uniform magnetic field of up to 20 mT must be generated over a 1.5 × 1.5 × 1.5 cm sample volume. The magnetic field was measured using a portable Gaussmeter (Model 5170, F.W. Bells, Magnetic Sciences Inc., Acton, MA, USA). Each coil must have a resistance of approximately 1 Ω to allow current control through spools with a 2 Ω load audio amplifier. The adjustable parameters included the wire gauge, inner and outer coil diameters, and coil thickness. Given the parameters, a MATLAB (The MathWorks, Inc., Natick, MA, USA) script was used to determine the coil dimensions for different-gauge square wires [26]. It was determined that a 20 gauge square magnetic wire (MWS Wire Industries, Westlake Village, CA, USA, part number M201171130) wound on a pair of small spools (an inner diameter of 3.4 cm, an outer diameter of 6.0 cm, and a width of 3.1 cm) and a pair of big (inner diameter of 3.4 cm, outer diameter of 8.5 cm, and width of 2.0 cm) spools satisfied all requirements. An appropriate support structure for the spools that could be inserted into the microscope was also designed. The spools and their supporting structure were 3D printed using versatile plastic (Shapeways, New York, NY, USA, Nylon 12 Plastic, PA12, Polyamide, PA 2200) with 100% infill. Spools were wound using a manual coil winding machine (U.S. Solid, Cleveland, OH, USA, NZ-2, UPC: 888107032434). Figure 1A shows the assembled coil insert, which was 13 cm long and 9.5 cm wide.

For the insert to rest at the correct height, a stage and legs were designed to hold it, as shown in Figure 1B. The posts and clips were obtained from Thorlabs, Inc. (Newton, NJ, USA). Nylon rods, an acrylic platform, and wing nuts to adjust the height of the platform holding the insert were obtained from McMaster-Carr (Elmhurst, IL, USA). The platform had an opening just large enough to have room for a 10×, 20×, or 40× objective. A sample holder to hold the magnetic particle clusters that were suspended in fluid for imaging was also designed and 3D printed. Figure 1C shows the entire coil assembly with the sample holder inserted into a Leica DMI3000 microscope.

### 2.4. Magnetic Particle Cluster Imaging

One pair of spools was used to produce an oscillating magnetic field. One function generator (DS345 Function Generator, Stanford Research Systems, Sunnyvale, CA, USA) was used in conjunction with the camera (Evolve Delta, Teledyne Photometrics, Tucson, AZ, USA), and μmanager’s “strobed” setting was employed to maintain a constant frame rate when imaging. μManager is an open-source microscopy software that controlled the microscope camera [27]. Another function generator (DG1000Z Series Waveform Generators model DG1032Z, Rigol, Beaverton, OR, USA) was used to drive the magnetic field at the desired frequency, outputting a 5 V peak-to-peak signal to an amplifier (MAGNITUDE2400, Seismic Audio, Memphis, TN, USA). The testing of particle cluster motion at magnetic field magnitudes lower than 2.5 mT required lower output voltage settings of the function generator. We applied an external magnetic field at 10 Hz; then, it ranged from 25 to 100 Hz, increasing in 25 Hz increments. This range overlapped with other studies investigating the magneto-mechanical action of nanoparticles in low-frequency alternating fields [23]. These fields are referred to as “non-heating alternating fields”, as their frequency is too low to induce heating in magnetic nanoparticles [22]. When imaging particle cluster rotation, the second coil pair was used in conjunction with the first pair, and a second channel on the function generator was connected to a second amplifier to run at the same settings, but 90 degrees out of phase, which could be achieved by applying a sine function to one channel and a cosine function to the other channel [7]. Due to each coil pair having different physical dimensions, the voltage from the function generator was amplified by a different magnitude for each amplifier to create an approximately equal magnetic field strength at the center of the sample holder from each coil set.

With the magnetic field applied to the particle clusters, μmanager was used in conjunction with the microscope stage and a 20× objective (Leica) to locate rotating or oscillating particle clusters, as well as to isolate a single particle cluster in a 64 × 64 pixel (0.8 μm per pixel) field of view for imaging. Keeping the field of view close to the actual particle cluster size allowed the use of higher imaging frame rates. For the experiments presented here, the camera frame rate was set to 300 Hz and the image exposure time was set to 0.7 ms. This allowed accurate data collection for particle cluster oscillation and rotation frequencies up to 150 Hz.

At each frequency, particle cluster motion was measured at various magnetic field strengths, decreasing steadily until oscillation or rotation could no longer be detected, and the minimum magnetic field strength at which particle cluster motion was observed was recorded. This was done with at least five different particle clusters at each frequency. The approximate sample temperature was recorded as well to calculate the viscosity of the fluid. The fluid temperature was estimated using a thermocouple (Bioptechs) that was placed above the sample, between the spools.

### 2.5. Image Analysis

**Figure 2:**
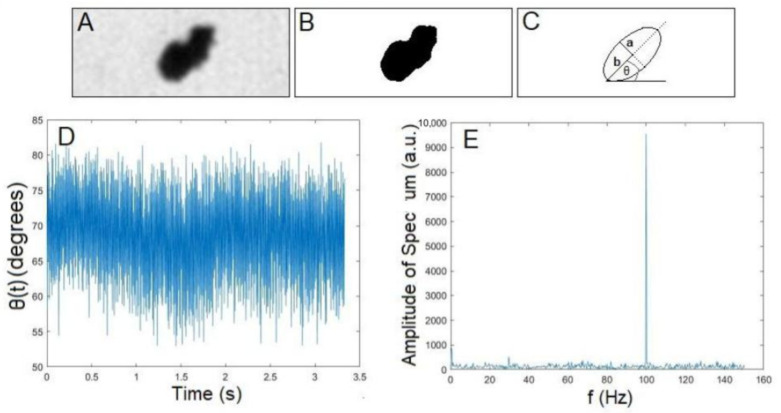
Sample image of an FeNP cluster, corresponding fitting of an ellipse in ImageJ, sample time series of an extracted particle cluster angle in ImageJ, and corresponding frequency spectrum obtained in MATLAB. (**A**) Filtered particle cluster image obtained from the camera. (**B**) Image in A after the thresholding used to estimate particle cluster size. (**C**) Ellipse fitted to the image in B after using the ImageJ “fit ellipse” function. (**C**) The angle θ between the major axis of the ellipse against the horizontal. The minor axis length *a* = 1.7 μm and major axis length *b* = 2.5 μm are also depicted. (**D**) θ(t) angle in time, θ(t), for an FeNP oscillating in glycerol driven at 100 Hz. (**E**) Corresponding frequency spectrum and measured oscillation peak at 100 Hz, obtained via a Fourier transform method.

For each isolated particle, 1000 images were taken 0.7 ms apart and saved as image stacks. Individual image stacks were analyzed using the ImageJ software [28], following the analysis presented in Reference [7]. Briefly, an ellipse was fit to each thresholded particle cluster image, with θ describing the angle between the horizontal image axis and the ellipse’s major axis; see Figure 2C. The program was also set to measure the area of the target particle cluster in μm${}^{2}$ using the ImageJ software; see the thresholded particle cluster image in Figure 2B. The particle cluster oscillation or rotational frequency was obtained by converting the particle cluster angle in time, θ(t) (see Figure 2D), to a frequency spectrum via Fourier transform methods; see Figure 2E. The highest peak in the frequency space was recorded as the measured frequency of oscillation or rotation. For rotational data, double-counting was taken into account. The lengths of the major and minor axes were used to estimate the dimensions of a rotating upright cylinder modeled in Section 2.6. Specifically, the minor axis length, *a*, was used as the radius of the cylinder, and the major axis length, *b*, multiplied by two was used as the height of the cylinder; see Figure 2C.

### 2.6. Theoretical Model

To calculate the minimum magnetic field needed to oscillate or rotate magnetic particle clusters in a given viscous fluid, we followed Reference [29]. In this model, a cylinder is rotated around the axis going along its height. In this simplified model, we assume that a single-domain magnetic particle is rotating perpendicularly to that direction. The magnitude of the torque *T* required to rotate a cylinder of radius $R_{i}$ and length *L* in a Newtonian fluid of viscosity μ is given as

(1) $$T=\frac{2\pi \mu L\omega R_{i}}{\frac{1}{R_{i}}-\frac{1}{R_{0}}}.$$

Water and glycerol viscosities at given temperatures were obtained from an online viscosity calculator [30]. Water’s viscosity ranges from 1.0 × 10${}^{-4}$ Pa·s at 26 ${}^{\circ }$C to 4.68 × 10${}^{-3}$ Pa·s at 60 ${}^{\circ }$C. Glycerol’s viscosity ranges from 1.41 Pa·s at 20 ${}^{\circ }$C to 0.08 Pa·s at 60 ${}^{\circ }$C. Here, $R_{0}$ is the radius of a cylindrical container in which the rotating cylinder resides, where $R_{0}>R_{i}$, and ω denotes the angular frequency of rotation. We note that the velocity profile of the fluid is assumed to be linear between $R_{0}$ and $R_{i}$. For our purposes, $R_{0}$ goes to infinity in Equation (1), $R_{i}$ is the average radius of the minor axis and major axis, i.e., $R_{i}=\frac{a+b}{2}$, of the two-dimensional ellipse, and *L* is twice the minor radius *a* of the ellipse, i.e., L=2a. The resulting equation for the magnitude of the torque needed to rotate the particle at a given frequency *f* is therefore

(2) $$T=2\pi^{2}\mu fa{\left(a+b\right)}^{2},$$

where ω=2πf. This torque must be supplied by the magnetic field produced by the wire spools. The magnitude of the magnetic torque is given as $\vec{m}\times \vec{B}$, where *m* is the magnetic moment of the particle and *B* in tesla is the magnetic flux density, which we call the magnetic field here. The magnetic moment can be found by using the magnetization *M* of the magnetic material, namely, m=MρV. In this work, magnetic particle clusters of randomly oriented multi-domain particles are considered as the magnetic entities. The size and direction of the magnetic moments of such clusters are unpredictable [31]. Hence, as a first estimate, we use the experimentally measured saturation magnetization of single-domain particles for *M* (see Section 3.1), with ρ being the density of the magnetic material and *V* the volume of the particles or, in this case, the volume of a cylinder of radius $\frac{a+b}{2}$ and length 2a. After setting *T* given in Equation (2) to mB, we can solve for the minimum magnetic field $B_{min}$ required to oscillate or rotate a magnetic particle in a given viscous fluid:

(3) $$B_{min}=\frac{4\pi \mu f}{M\rho }.$$

**Figure 3:**
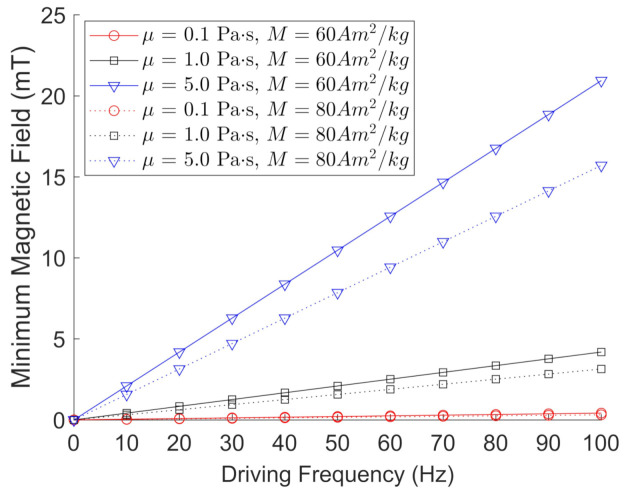
Calculated values using Equation (3) for the minimum magnetic field required to oscillate/rotate magnetic particles with ρ = 5.00 g/cm${}^{3}$ and *M* = 60 Am${}^{2}$/kg (solid lines) or *M* = 80 Am${}^{2}$/kg (dotted lines) ranging from 10 Hz to 100 Hz, and fluid viscosities ranging from 0.1 Pa·s to 5.0 Pa·s.

Here, we use ρ = 5.00 g/cm${}^{3}$ for the FeNPs and ρ = 5.28 g/cm${}^{3}$ for the BaPs. We note that $B_{min}$ does not depend on the dimensions of the particle, which is a shortcoming of this simplified model. Figure 3 depicts the calculated values for the minimum magnetic field needed to oscillate/rotate magnetic particles with ρ = 5.00 g/cm${}^{3}$ and *M* = 60 Am${}^{2}$/kg (solid lines) or *M* = 80 Am${}^{2}$/kg (dotted lines) under a given frequency and viscosity. All graphs exhibited a linear trend, and the minimum magnetic field $B_{min}$ required to oscillate or rotate a magnetic particle decreased with increasing particle magnetization, as expected.

## 3. Results

### 3.1. Magnetic Particle Characterization

**Figure 4:**
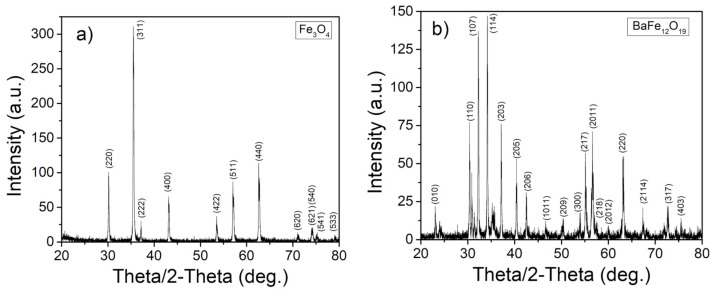
XRD patterns for (**a**) Fe${}_{3}$O${}_{4}$ and (**b**) BaFe${}_{12}$O${}_{19}$.

Before the particle motion experiments were performed, the particles’ crystallographic structure, magnetization, and size were characterized. First, both types of magnetic particles were characterized through Bragg–Brentano geometry XRD to verify the crystallographic structure and purity of the particle powders. Figure 4a shows the strong characteristic peaks of single-phase iron oxide, Fe${}_{3}$O${}_{4}$ [32]. The characteristic peaks of barium hexaferrite, BaFe${}_{12}$O${}_{19}$, are readily apparent in Figure 4b [33].

**Figure 5:**
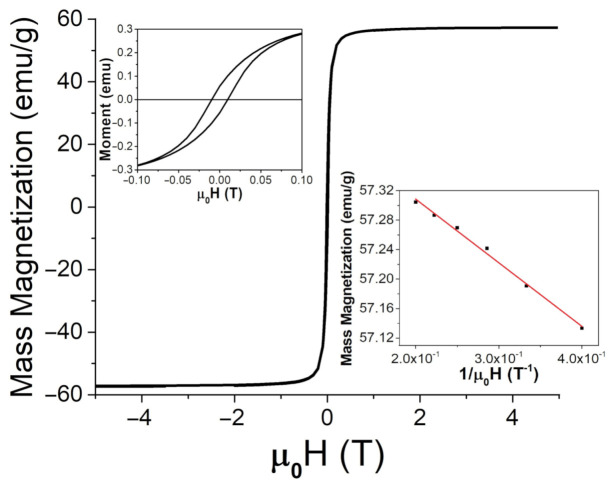
SQUID measurements for Fe${}_{3}$O${}_{4}$ at room temperature (26 ${}^{\circ }$C or 298 K) up to 5 T, with an enlarged region for lower applied fields (upper insert) and a comparison between the magnetic moment and $1/\mu_{0}$H at high applied fields to determine the saturation magnetization from Equation (4), $M_{S}$ (lower insert).

**Figure 6:**
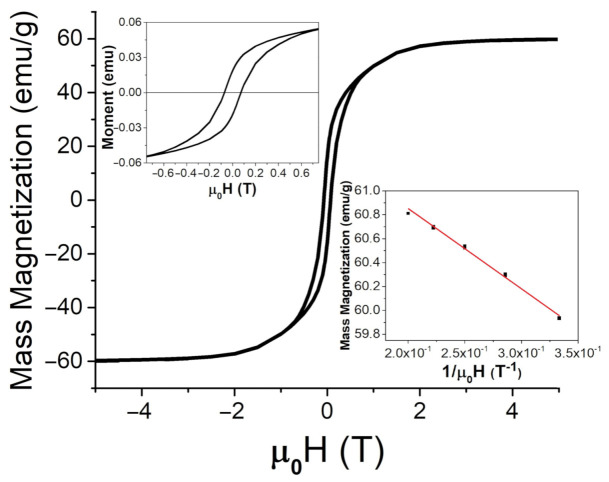
SQUID measurements for BaFe${}_{12}$O${}_{19}$ at room temperature (26 ${}^{\circ }$C or 298 K) up to 5 T, with an enlarged region for lower applied fields (upper insert) and a comparison between the magnetic moment and $1/\mu_{0}$H at high applied fields to determine the saturation magnetization from Equation (4), $M_{S}$ (lower insert).

Magnetic characterization of the particles was performed by employing a SQUID magnetometer. The hysteresis loops for both types of particle powders can be seen in Figure 5 and Figure 6. The applied magnetic field, $\mu_{0}H$, was swept between ±5 T. Saturation magnetization, $M_{S}$, was found using the law of approach to saturation [34], written as

(4) $$M=M_{S}\left(1-a/H-b/H^{2}-\ldots \right).$$

Here, *a* and *b* are fitting constants. At room temperature (26 ${}^{\circ }$C or 298 K), mass magnetization was found to be 57.5 ± 0.1 emu/g (or 57.5 ± 0.1 Am${}^{2}$/kg) and 62 ± 1 emu/g (or 62 ± 1 Am${}^{2}$/kg) for the FeNPs and BaPs, respectively. The coercivity of the FeNPs was determined to be 9.6 mT, and it was 72 mT for the BaPs. These results indicate significantly different strengths of anisotropy fields in these two types of particles.

**Figure 7:**
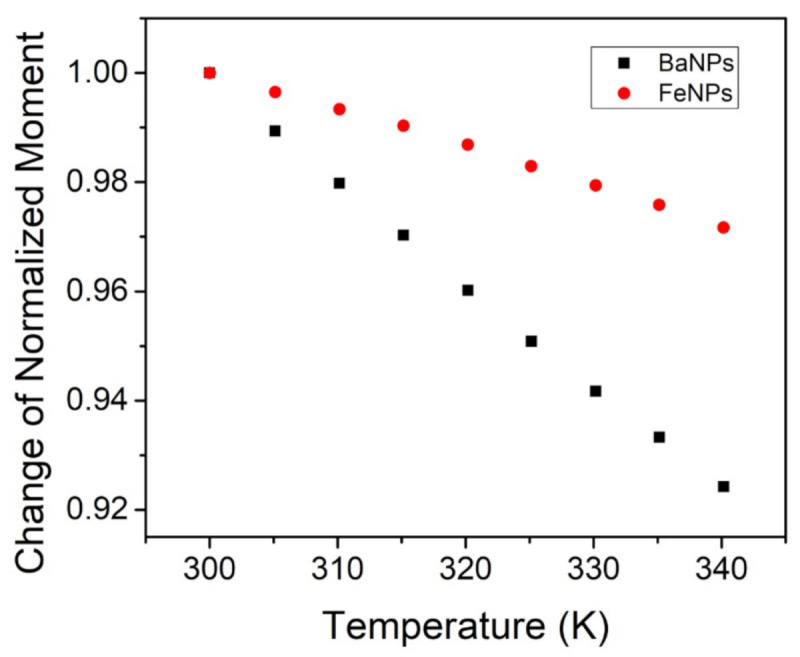
Normalized SQUID measurements of BaFe${}_{12}$O${}_{19}$ (black) and Fe${}_{3}$O${}_{4}$ (red) with respect to magnetic moment and temperature at a constant 1 T applied field.

Measurements of the temperature dependence of magnetization were performed with a constant applied external field at 1 T; see Figure 7. The FeNPs exhibited a minimal reduction in the magnetic moment between room temperature (26 ${}^{\circ }$C or 298 K) and 57 ${}^{\circ }$C (or 330 K): approximately 2%. The BaPs experienced a reduction in the moment of about 6% over the same temperature range. Saturation magnetization is known to decrease with increasing temperature due to thermal disorder, which increases with temperature and hinders magnetic dipoles in aligning with the applied magnetic field [31,35,36].

**Figure 8:**
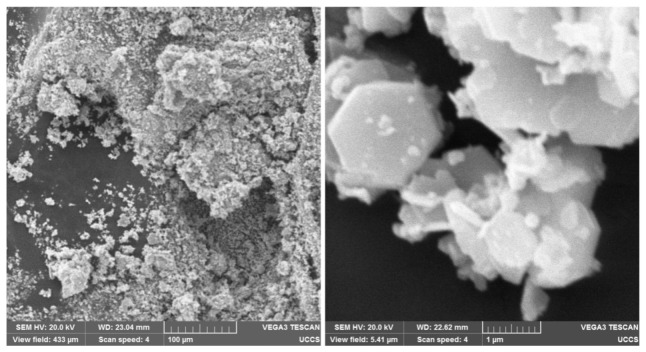
SEM images of BaFe${}_{12}$O${}_{19}$ particles and the clusters that they form. On the left, magnification is 500×. On the right, magnification is 40,000×.

**Figure 9:**
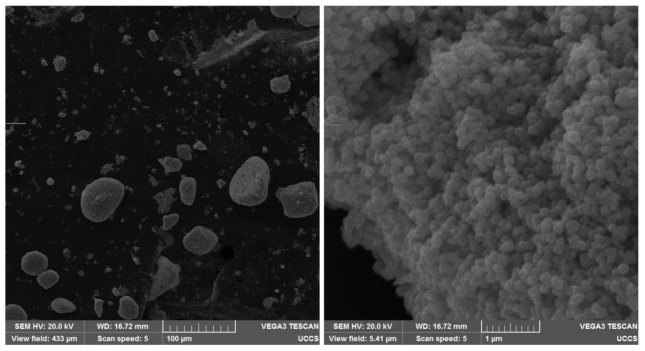
SEM images of Fe${}_{3}$O${}_{4}$ nanoparticles and the clusters that they form. On the left, magnification is 500×. On the right, magnification is 40,000×.

SEM images of the BaFe${}_{12}$O${}_{19}$ and Fe${}_{3}$O${}_{4}$ particle clusters are presented in Figure 8 and Figure 9, respectively. A magnification of 40,000× (see the right-hand pictures) depicts the hexagonal structure of the BaFe${}_{12}$O${}_{19}$ particles [37] and roundish shape of the Fe${}_{3}$O${}_{4}$ nanoparticles [38]. Individual Fe${}_{3}$O${}_{4}$ nanoparticles were more homogeneous in size than individual BaFe${}_{12}$O${}_{19}$ particles, which were polydisperse hexagonal flakes, according to their datasheets. The SEM images depict that the particle clusters consisted of many individual particles, indicating that the presented work characterized the motion of multi-domain particles in the fluids of different viscosities.

### 3.2. Minimum Magnetic Field Measurements

**Figure 10:**
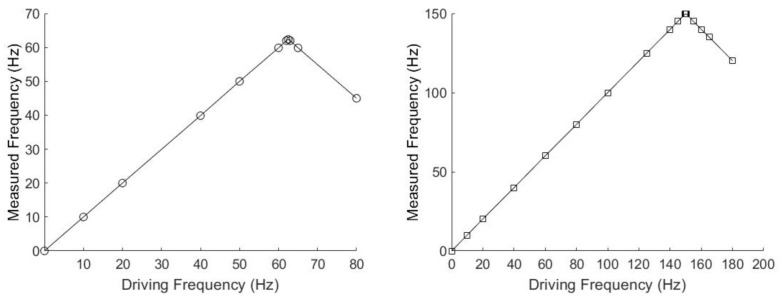
Driving frequencies compared to the experimentally measured frequencies for BaP clusters in water. These data were taken at frame rates of 125 Hz (**left**) and 300 Hz (**right**).

In the experiments presented here, magnetic particle clusters were subjected to low driving frequencies ranging from 10 to 180 Hz to accommodate the camera frame rate and, hence, visualize individual particle cluster oscillation and rotation using a light microscope. In general, among all of the set camera frame rates, we were able to get accurate results with various frequencies that ranged from 10 Hz to half the set camera frame rate. For example, for BaP clusters rotating in water, if the camera frame rate was set to 125 Hz, we were able to measure particle cluster oscillation/rotation in water up to 62.5 Hz; see the left panel of Figure 10. At a camera frame rate of 300 Hz, we were able to measure the rotation/oscillation frequencies of the particle clusters up to 150 Hz in water; see the right panel of Figure 10. When the driving frequencies were set larger than half the set camera frame rate, the measured rotation/oscillation frequencies were lower than the driving frequencies in water, as follows from the sharp change in slope in Figure 10, as expected with the Nyquist–Shannon sampling theorem [39]. The camera allowed for a maximum frame rate of 381 Hz. Increasing the frame rate decreased the exposure time and, hence, reduced the contrast of the imaged particle clusters. Up to a 300 Hz frame rate, the contrast of the particle cluster images was acceptable; above 300 Hz, our analysis method was less accurate due to the inability to sufficiently differentiate between clusters and the image background. For particle clusters rotating in water, we were not able to experimentally verify the minimum required magnetic field obtained from Equation (3). For example, to rotate a BaP or a, FeNP in water at 10 Hz with a sample temperature of 23 ${}^{\circ }$C, the minimum magnetic fields obtained from Equation (3) were 3.4 × 10${}^{-4}$ mT and 3.9 × 10${}^{-4}$ mT, respectively. These magnetic field magnitudes were too small to be investigated with the available equipment. Table 1 shows the average measured cluster size in the form of imaged particle cluster area +/− the standard deviation of oscillating and rotating FeNP and BaP clusters at different frequencies in water. The cross-sectional area of the particle clusters ranged from 1 to 964 μm${}^{2}$, showing that FeNPs and BaPs typically form large clusters in the micron-size range in water. Table 2 depicts the corresponding ranges of particle cluster diameters, assuming the fitted ellipse area to be a circle. The particle cluster diameter ranged from 5 to 45 μm.

**Figure 11:**
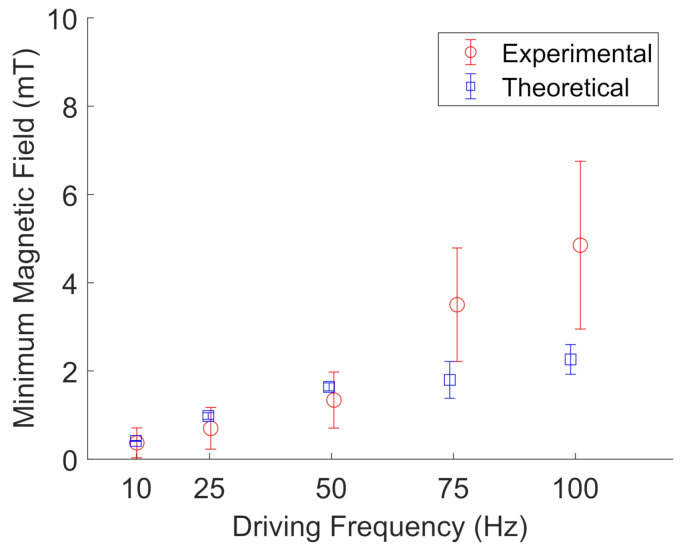
Experimental (red, open circles) measurements of the minimum magnetic field needed to oscillate BaP clusters in glycerol compared to their theoretical values (blue, open squares) at a given driving frequency. Error bars represent the standard deviation of five different particle clusters. Data points are slightly offset along the x-axis to aid visualization.

**Figure 12:**
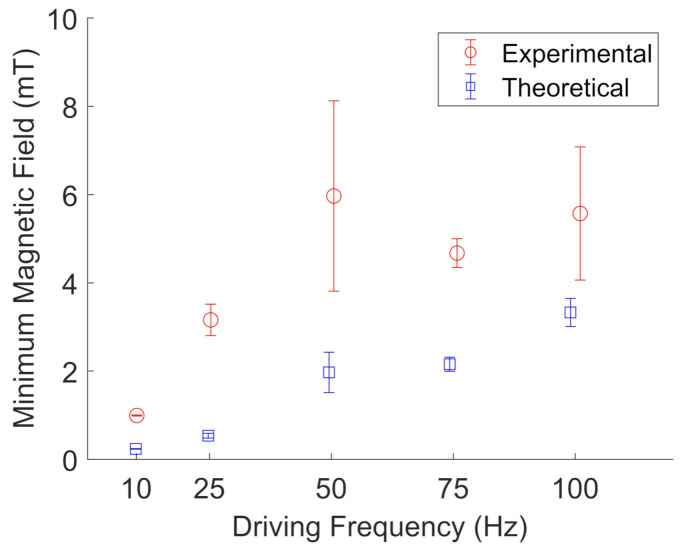
Experimental (red, open circles) measurements of the minimum magnetic field needed to oscillate FeNP clusters in glycerol compared to their theoretical values (blue, open squares) at a given driving frequency. The error bars represent the standard deviations of five different particle clusters. The data points are slightly offset along the x-axis to aid visualization.

Figure 11 and Figure 12 show the results for BaP and FeNP cluster oscillation in glycerol, respectively. The open blue squares represent the theoretical calculations by evaluating Equation (3) for the determined viscosity and magnetization corresponding to the estimated sample temperature. The viscosity was obtained from an online viscosity calculator [30]. The magnetization was determined from Figure 7. The open red circles represent the experimentally measured frequencies. The error bars represent the standard deviation of five different particle clusters. For both BaP cluster and FeNP cluster oscillation in glycerol, the experimental results are in qualitative agreement with the proposed model, with an average deviation from the model of 1.7 ± 1.3.

**Figure 13:**
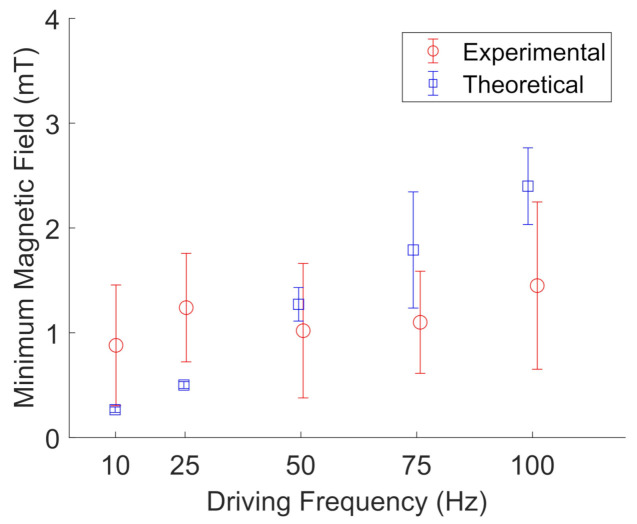
Experimental (red, open circles) measurements of the minimum magnetic field needed to rotate BaP clusters in glycerol compared to their theoretical values (blue, open squares) at a given driving frequency. The error bars represent the standard deviations of five different particle clusters. The data points are slightly offset along the x-axis to aid visualization.

**Figure 14:**
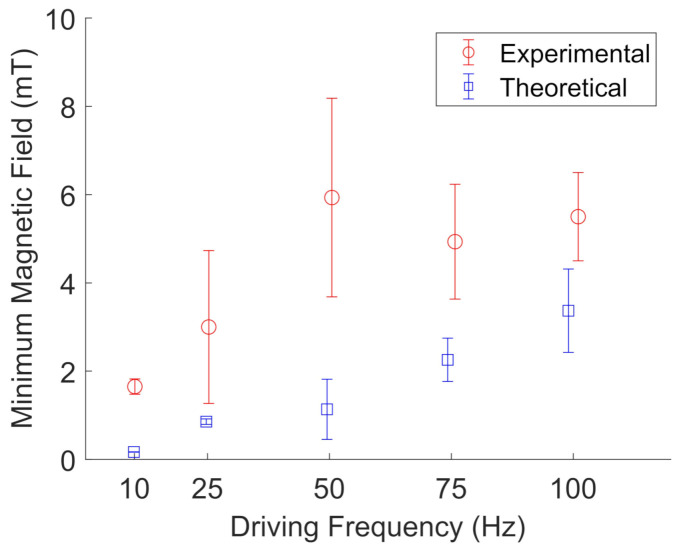
Experimental (red, open circles) measurements of the minimum magnetic field needed to rotate FeNP clusters in glycerol compared to their theoretical values (blue, open squares) at a given driving frequency. The error bars represent the standard deviations of three different particle clusters. The data points are slightly offset along the x-axis to aid visualization.

Figure 13 and Figure 14 show the results for particle cluster rotation in glycerol when two pairs of wire spools were used to apply a rotating magnetic field. The error bars represent the standard deviation of five different particle clusters. In glycerol, the experimental results are in qualitative agreement with the proposed model for particle cluster rotation, with an average deviation from the model of 1.6 ± 1.4. Table 3 shows the average measured particle cluster size in the form of the imaged particle cluster area +/− the standard deviation of oscillating and rotating FeNP and BaP clusters at different driving frequencies in glycerol. The cross-sectional area of the particle clusters ranged from 20 to 627 μm${}^{2}$, indicating large-scale NP clustering, as observed in water. The particle cluster diameter ranged from 6 to 35 μm; see Table 4. Furthermore, a wider range of different particle cluster sizes was able to rotate or oscillate in glycerol. We noted that it was unusually difficult to measure accurate rotating frequencies for FeNP clusters in glycerol. Hence, the error bars are based on the standard deviation of three data points instead of five, as fewer particle clusters were imaged.

## 4. Discussion

Magnetic particles can travel through highly viscous fluids when applying a strong enough magnetic field to influence them. In this paper, we showed that the experimentally measured minimum magnetic field needed to oscillate or rotate multi-domain magnetic BaFe${}_{12}$O${}_{19}$ and Fe${}_{3}$O${}_{4}$ particle clusters in glycerol at a given driving frequency was within a factor of five when compared to the predictions of a simplified model for single-domain magnetic particles (given in Equation (3)). This model is independent of particle shape. The theoretical calculations assumed that the particles were perfectly smooth cylinders isolated in the solution. In the actual experiments, the particle clusters were irregularly shaped and composed of magnetic multi-domains. The former potentially resulted in higher fluid drag than that which was modeled, and the latter resulted in a lower magnetization value than that used [31]. Additionally, the mathematical model did not model particle–surface or particle–particle interactions. Furthermore, magnetic anisotropy, which is known to affect the physical properties of barium hexaferrite, was not included in our simplified model [41]. Moreover, the actual temperature of the fluid was likely lower than the temperature used to evaluate Equation (3). This is due to the physical placement of the thermistor outside the sample chamber, likely resulting in an underestimation of the fluid viscosities and, therefore, overestimation of the minimum magnetic fields. Specifically, the generated heat from the spools likely did not have sufficient time to be fully transferred through the glass of the sample slide and, hence, to the fluid by the time images were collected. For example, at magnetic field magnitudes of around 8 mT, the current-carrying wire spools heated up from room temperature to about 40 ${}^{\circ }$C within a few minutes. Future research will try to address the outlined shortcomings of the presented model.

## 5. Conclusions

The purpose of this project was to study the oscillation and rotation of BaFe${}_{12}$O${}_{19}$ and Fe${}_{3}$O${}_{4}$ magnetic particle clusters in a fluid of significantly higher viscosity than water. In this work, we showed that the simplified model given in Equation (3) can estimate the minimum required magnetic field to rotate/oscillate magnetic particle clusters of diameters ranging from 5 to 45 μm in viscous fluids with viscosities up to 1.4 Pa·s within less than an order of magnitude. Specifically, the experimental results are in qualitative agreement with the proposed model, with an average deviation from the model of 1.7 ± 1.3 for oscillation and rotation frequencies ranging from 10 to 180 Hz and magnetic field magnitudes ranging from 0.25 to 9 mT. Hence, Equation (3) may be a useful approximation for future research in this area. Biomedical applications based on moving magnetic particles through biological fluids with the help of magnetic fields can be used for targeting and destroying cancer cells [18,19,20,21] and drug delivery [6] through respiratory mucus in order to treat asthma and other lung diseases [4,5]. When applying this technique for drug delivery through mucus to deceased airway epithelial cells, the magnetic field will need to be significantly higher than that used in this study, at least in the 200 to 700 mT range [14,19]. The results presented here can, therefore, be used as a springboard for future investigations in the field of magnetic-particle-aided drug delivery through high-viscosity fluids.

## Figures and Tables

**Table 1:** The average measured particle cluster area +/− the standard deviation of oscillating and rotating FeNPs and BaPs at different frequencies in water for 10, 20, 40, 60, and 100 Hz. Corresponding data for 80 Hz are not shown, as they correlated with the other frequencies.

Particle Cluster Area (μm${}^{2}$)	10 Hz	20 Hz	40 Hz	60 Hz	100 Hz
BaP Oscillation	225 +/− 345	220 +/− 273	420 +/− 297	453 +/− 236	369 +/− 254
FeNP Oscillation	42 +/− 22	160 +/− 57	169 +/− 38	78 +/− 59	105 +/− 43
BaP Rotation	392 +/− 289	594 +/− 220	258 +/− 145	360 +/− 245	359 +/− 417
FeNP Rotation	568 +/− 280	826 +/− 138	375 +/− 161	218 +/− 61	162 +/− 161

**Table 2:** The average measured particle cluster diameter of oscillating and rotating FeNPs and BaPs at different frequencies in water for 10, 20, 40, 60, and 100 Hz, corresponding to the average area given in Table 1. The cluster diameter was calculated assuming the ellipse area to be a circle, $d=2\sqrt{\frac{A}{\pi }}$, where *A* is the area and *d* is the diameter, +/− error propagation from the propagation of the uncertainty variance formula, as outlined in Reference [40].

Particle Cluster Diameter (μm)	10 Hz	20 Hz	40 Hz	60 Hz	100 Hz
BaP Oscillation	17.3 +/− 7.7	22.9 +/− 4.6	24.2 +/− 11.2	23.4 +/− 11.2	23.4 +/− 11.2
FeNP Oscillation	7.3 +/− 1.9	14.7 +/− 2.5	14.7 +/− 1.7	9.9 +/− 3.8	11.6 +/− 2.3
BaP Rotation	21.6 +/− 13.6	22.7 +/− 11.1	25.5 +/− 7.2	20.7 +/− 8.2	32.2 +/− 10.3
FeNP Rotation	26.9 +/− 6.6	32.4 +/− 2.7	21.6 +/− 7.8	16.3 +/− 18.1	14.4 +/− 7.1

**Table 3:** The average measured particle cluster area +/− the standard deviation for FeNP and BaP cluster oscillation and rotation at different driving frequencies in glycerol.

Particle Cluster Area (μm${}^{2}$)	10 Hz	25 Hz	50 Hz	75 Hz	100 Hz
BaP Oscillation	273 +/− 250	426 +/− 174	537 +/− 407	509 +/− 432	516 +/− 442
FeNP Oscillation	398 +/− 229	187 +/− 85	51 +/− 3	183 +/− 127	51 +/− 2
BaP Rotation	82 +/− 62	320 +/− 11	315 +/− 9	181 +/− 81	154 +/− 34
FeNP Rotation	400 +/− 10	349 +/− 33	464 +/− 82	433 +/− 2	452 +/− 4

**Table 4:** The average measured particle cluster diameter of oscillating and rotating FeNPs and BaPs at different frequencies in glycerol for 10, 20, 40, 60, and 100 Hz, corresponding to the average area given in Table 3. The cluster diameter was calculated assuming the fitted ellipse area to be a circle, $d=2\sqrt{\frac{A}{\pi }}$, where *A* is the area and *d* is the diameter, with +/− error propagation from the propagation of uncertainty variance formula, as outlined in Reference [40].

Particle Cluster Diameter (μm)	10 Hz	20 Hz	40 Hz	60 Hz	100 Hz
BaP Oscillation	17.3 +/− 7.7	22.9 +/− 4.6	24.2 +/− 11.2	23.4 +/− 11.2	23.4 +/− 11.2
FeNP Oscillation	22.5 +/− 6.5	15.4 +/− 3.5	18.7 +/− 4.9	15.2 +/− 5.3	8.1 +/− 0.2
BaP Rotation	10.2 +/− 3.9	20.2 +/− 0.3	20.0 +/− 0.3	15.2 +/− 3.4	14.0 +/− 1.5
FeNP Rotation	22.6 +/− 0.3	21.1 +/− 1.0	24.3 +/− 2.1	23.4 +/− 0.1	24.0 +/− 0.1

## Data Availability

Not applicable.
